# Epidemiological aspects of individuals with mental disorders in the referral system: the experience of a Community Mental Health Center in the northeast of Iran

**DOI:** 10.1007/s44192-024-00078-1

**Published:** 2024-06-21

**Authors:** Mahdi Talebi, Shabnam Niroumand, Mobin Gholami, Azadeh Samarghandi, Fatemeh Shaygani, Mahdi Radfar, Ahmad Nemati

**Affiliations:** 1 Department of Community and Family Medicine, Faculty of Medicine, Mashhad University of Medical Sciences, Mashhad, Iran; 2 Faculty of Medicine, Mashhad University of Medical Sciences, Mashhad, Iran; 3 Department of Psychology, Faculty of Education Sciences and Psychology, Ferdowsi University of Mashhad, Mashhad, Iran; 4 Student Research Committee, Shiraz University of Medical Sciences, Shiraz, Iran

**Keywords:** Community Mental Health Centers, Mental health, Epidemiology, Referral and consultation, Patient dropouts, Iran

## Abstract

**Background:**

Community Mental Health Centers (CMHCs) offer affordable mental health services in a less stigmatized environment, in a domiciliary setting. This study aimed to shed light on the epidemiological factors of patients attending CMHCs of Mashhad, their referral status, and treatment.

**Methods:**

This study was conducted over the medical records of patients seen by psychiatrists between January 2014 and December 2021 in Mashhad's CMHC, the northeast of Iran. A detailed questionnaire was used to extract data from medical records about the epidemiological characteristics, diagnosed mental illnesses, referral status, and how often they visited the psychiatrist. The association between epidemiological findings and patient referral (referral system or self-referral) as well as the association between epidemiological findings and the number of psychiatric revisits were examined using the Chi-square test.

**Results:**

Out of 662 patients, 472 (71%) were female and 190 (29%) were male, with an average age of 29 years. Among the 475 adult patients, 367 (77.3%) were married, with the majority being homemakers (56.4%). Major Depression Disorder (MDD) (32%) and Generalized Anxiety Disorder (GAD) (18.3%) were the most prevalent mental health conditions among patients. The majority of patients (74.9%) were referred to the CMHC of Mashhad from Primary Healthcare centers (PHCs) and psychiatric hospitals. Furthermore, female gender and patients with lower level of education were associated with more referral through from referral system. Of note, 431 patients (65.1%) did not return for a second visit, the ratio of treatment dropout was higher for patients with lower education levels.

**Conclusions:**

Referral system should be more practical in Iran to enhance health services in CMHCs. It is recommended that PHCs undergo certain modifications to enhance the referral process for patients with mental health conditions, focusing on common mental disorders and individuals with low socioeconomic level.

## Background

Mental disorders are widespread in all countries; the World Health Organization (WHO) stated that in 2019 almost one in eight people suffered from mental disorders [1]. Mental disorders are also a significant health problem in Iran; according to a 2015 study, the prevalence rate of mental disorders is around 23%. Also, mental disorders are among the most important contributors to the burden of disease and disability-adjusted life years (DALYs) in Iran [2, 3]. Recent research indicates that there is a higher occurrence of mental illness in Iranian population, for example in 2019, 37% of people in Tehran, the capital city of Iran had mental health conditions [4]. A population-based study in Ilam, Iran reported that 19.9% of individuals experienced depression while 25.9% of them experienced anxiety [5].

In the history of the development of psychiatric care worldwide, various models have been developed. The Community Mental Health Center (CMHC) stands out as a model that offers mental health services to patients at a reduced cost, with decreased stigma, in a domiciliary setting, and with easy accessibility [6, 7]. The main goal of CMHC is to facilitate outpatient mental health treatment. In this model, patients are referred from two distinct settings: primary healthcare centers (PHCs) and psychiatric hospitals. PHCs serve as the initial point of contact for individuals within the healthcare system and can play a vital role in reducing the expenses associated with treating patients with mental health conditions by providing early screening, effective treatment, and referrals to specialized CMHCs [8, 9]. In addition, patients with previous psychiatric admissions, most of whom have severe mental illness, are referred to CMHC for treatment and follow-up. The referral in healthcare system offers accessible, efficient, and fair healthcare for all individuals [10]. The term "Self-referral" is used to describe patients who utilize the services of a higher level of the healthcare system without any referral from lower levels of healthcare system [11]. Treatment dropout among patients with mental health conditions is a significant issue, with rates between 20 and 60% globally [12]. In Iran, a study showed that 57% of outpatient psychiatric patients in CMHCs discontinued treatment [13].

Iran's first Community Mental Health Center (CMHC) was established in Tehran in 2010. The Iranian CMHC model, designed using research data, collaborative care and aftercare in day centers. Collaborative care involves general practitioners (GPs) in primary health care centers (PHCs), treating and identifying patients with non-severe mental illnesses and referring them to CMHCs if needed. Aftercare focuses on treatment and follow-up for patients with severe mental illnesses, including those with a history of psychiatric hospitalization, and offers home care and telephone follow-up. CMHCs also provide mental health education for patients and their families [14–17]. Patients typically come to CMHCs via referral from PHCs or psychiatric hospitals. However, CMHCs are also accessible without a referral, and both referred and self-referred patients receive the same care, costs, and insurance coverage [18]. It should be noted that, there has been a lack of investigation into the adherence to the referral system, epidemiological factors and treatment dropout rates among patients with mental health conditions at the CMHCs of Iran. This information could provide valuable insights for health policy makers in Iran and other developing countries regarding the CMHC and the characteristics of its patient population and referrals in mental health services.

In 2014, the Mashhad University of Medical Sciences established the CMHC in Mashhad, Iran's second-largest city. It offers comprehensive mental health care for severe and non-severe illnesses, providing a beacon of hope in the field. Services include psychotherapy, pharmacotherapy, psychoeducation, cognitive rehabilitation, social meetings, and telephone follow-up provided by psychiatrists, psychologists, and trained nurses. This study is the first to analyze the patients at the Mashhad’s CMHC, offering insights into the referral system and treatment dropout rates among patients with mental health conditions and paving the way for further improvements. `

## Methods

This cross-sectional study was conducted between January 2014 and December 2021 at Mashhad's community mental health center (CMHC), northeast of Iran.

### Ethical issues

The study was approved by the Ethics Committee of the Research Department of Mashhad University of Medical Sciences, and the code was IR.MUMS.MEDICAL.REC.1399.701. Also, to collect data, we used patient clinical record codes instead of patient names to protect participants' privacy. Moreover, only clinical records containing signed consent forms for probable future studies were entered in this study.

### Data collection

Patient information and data collection involved reviewing all complete clinical paper records of patients visited by the psychiatrists at Mashhad’s CMHC. One challenge we encountered was the limited availability of clinical records that met the study's checklist criteria. Psychiatrists filled in details regarding patients' history of mental illness diagnosis, treatment, and revisits, while demographic characteristics and referral information were filled in by other staff members at Mashhad CMHC. To address this issue, we took two steps to select eligible medical records for the study. First, we included records with a definite diagnosis of mental illness and a recorded number of revisits. Then, we selected records that had at least 80% compliance with demographic variables and referral status.

We designed and used a checklist to extract demographic information, definite diagnosis of psychological disorders (based on DSM-4 criteria), number of revisits, and referral details from medical records. Since there are differences between common mental disorders in adults and children, we evaluated adults and children separately. The education, marital status, and employment rate of patients under 17 were not investigated. Additionally, a large proportion of child patients were in preschool ages. Also, clinical records that contained at least 80% of the data related to demographic characteristics and referral status was entered in the study. As a result, it should be cautioned that the total number of data in each category may be different.

### Statistical analysis

After reviewing the data obtained in the study checklist, these were entered into SPSS software version 11.5 and converted to nominal data. The frequency distributions resulting from demographic characteristics, referral, mental disorder, and the number of psychiatric visits were presented in the tables. An analysis was performed to examine the association between epidemiological findings and patient referral (referral system or self-referral) and the association of epidemiological findings and the number of psychiatric revisits; In this part, the Chi-square test was used. The level of significance was set at a p < 0.05.

## Results

**Fig. 1:**
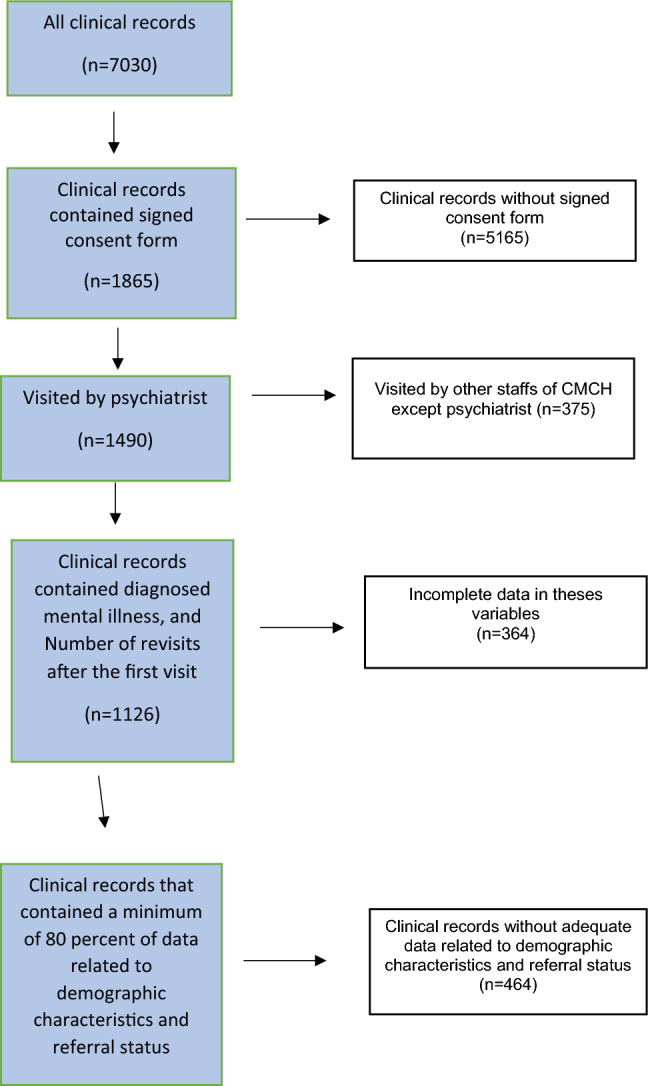
Flowchart of case selection

As revealed in Fig. 1, it was found that 662 patients met the eligibility criteria for inclusion in the study. The Fig. 1 outlining the process of inclusion and exclusion is shown at each stage in the flowchart.

472 (71%) women and 190 (29%) men were included in this study. The mean age of patients was 29 and most were in the 18 to 39 age group (44%). Meanwhile, 187 (28.2%) patients were children (17 or under 17). Among 475 adult patients, 367 (77.3%) patients were married. Table 1 shows the main characteristics of the patients.

**Table 1:** Main characteristics information of patients

Variable	Frequency (%)	Variable	Frequency (%)
Gender		Education	
Male	192 (29)	Illiterate	34 (8.2)
Female	470 (71)	Elementary and secondary	168 (40.4)
Age		Diploma or above diploma	153 (36.8)
0–17	187 (28.2)	Bachelor or above	61 (14.7)
18–39	291 (44)	Occupation	
40–59	150 (22.7)	Housewife	237 (56.4)
60 ≤	34 (5.1)	Unemployed	50 (11.9)
Marital status		Self-employment	46 (11)
Married	367 (77.3)	Government employee	32 (7.6)
Single	50 (10.5)	Worker	44 (10.5)
Widowed	24 (5.1)	Student	11 (2.6)
Divorced	34 (7.2)		

Table 2, presents an overview of mental illnesses, referral status, and the number of patient visits and compares these variables in children and adults. Because of the broad psychiatric diagnosis, we classified these patients into the group named "Other" with a frequency of fewer than 10 patients. Major Depression Disorder (MDD) (32%), Generalized Anxiety Disorder (GAD) (18.3%), and Attention Deficit Hyperactivity Disorder (ADHD) were the three most common mental illnesses. In adults, the three most common psychiatric disorders were MDD (42.5%), GAD (18.3%), and BMD (Bipolar Mood Disorder) (11.6%), and in children, ADHD (33%), GAD (18.2%), and ODD (Oppositional Defiant Disorder) (8%).

**Table 2:** Primary diagnosis of the patients and the referral methods to CMHC

Variable	Frequency (%)	Adult (%)	Children (%)
The primary diagnosis of the patients	662	475	187
GAD*	121 (18.3)	87 (18.3)	34 (18.2)
ADHD*	63 (9.5)	–	63 (33.7)
BMD*	55 (8.3)	55 (11.6)	–
MDD*	212 (32)	202 (42.5)	10 (5.3)
AD*	50 (7.6)	50 (10.5)	–
OCD*	26 (3.9)	19 (4)	7 (3.7)
Schizophrenia	15 (2.3)	15 (3.2)	–
ODD*	15 (2.3)	–	15 (8)
ED*	13 (2)	–	13 (7)
ASD*	10 (1.5)	–	10 (5.3)
Other	55(8.3)	27 (5.7)	28 (15)
Healthy	27 (4.1)	20 (4.2)	7 (3.7)
Referral methods	638	461	177
Referred from PHCs	451 (70.7)	334 (72.5)	117 (66.1)
Referred from psychiatric hospitals	27 (4.2)	23 (4.9)	4 (2.3)
Self-referred	160 (25.1)	104 (22.6)	56 (31.6)
Number of visits after the first visit	662	475	187
No revisit	431 (65.1)	309 (65.1)	122 (65.2)
1–2	113 (17.1)	80 (16.8)	33 (17.6)
3 ≤	118 (17.8)	80 (18.1)	32 (17.1)

^*^GAD: Generalized Anxiety Disorder, *ADHD*: Attention Deficit Hyperactivity Disorder, *BMD*: Bipolar disorder, *MDD*: Major Depression Disorder, *AD:* Adjustment Disorder, *OCD*: Obsessive–Compulsive Disorder, *ODD*: Oppositional Defiant Disorder, *ED*: Elimination Disorder, *ASD*: Autistic Specterum Disorder

As can be seen from Table 2, most patients (70.7%) were referred to this center through the Primary Healthcare Centers (PHCs), and 4.2% of patients were referred from psychiatric hospitals while psychiatrists visited 25.1% of patients without referral paper. An unexpected finding was that 431 (65.1%) patients did not return for a second visit, and only 118 (17.8%) patients had three or more revisits.

The following section of the survey focused on the association between demographics and referral from healthcare institutions including PHCs and psychiatric hospitals. According to the data presented in Table 3, there was a significant difference in the referral patterns to Mashhad's CMHC through the referral system based on the patients' education levels. Patients with lower education levels (including illiterates, primary, and secondary education) had a higher percentage of referrals (84.3% and 78.5%) compared to patients with higher education levels (diploma or above diploma, bachelor or above) (75% and 61.6%) (P = 0.04). Additionally, female patients were referred more frequently through the referral system compared to male patients (77.3% vs. 68.8%) (P = 0.024). Furthermore, patients who had 1–2 revisits were significantly more likely to be referred from the healthcare system (83.6%) compared to patients with more than 3 revisits or no visits (71.9% and 70.9%) (P = 0.035).

**Table 3:** Factors associated with referral status

Variable	Referral from PHCs or referred from psychiatric hospitals N (%)	Self-referral N (%)	P-value*
Gender			
Male	126 (68.8)	57 (31.2)	0.024*
Female	352 (77.3)	103(22.7)	
Age			
0–17	131 (74)	46 (26)	0.83
18–39	218 (76.4)	67 (23.6)	
40–59	106 (73.6)	38 (26.4)	
60 ≤	23 (71.8)	9 (28.2)	
Marital status			
Married	270 (76.2)	84 (23.8)	0.179
Single	33 (66)	17 (34)	
Widowed	18 (78.2)	5 (21.8)	
Divorced	29 (85.2)	5 (14.8)	
Education			
Illiterate	27 (84.3)	5 (15.7)	0.04*
Elementary and secondary	128 (78.5)	35 (21.5)	
Diploma or above diploma	111 (75)	37 (25)	
Bachelor or above	37 (61.6)	23 (38.4)	
Occupation			
Housewife and unemployed individuals	228 (82)	50 (18)	0.066
Student	8 (72.7)	3 (27.3)	
Self-employment	30 (65.2)	16 (34.8)	
Worker	32 (74.4)	11 (25.6)	
Government employee	18 (58)	13 (42)	
Number of visits after the first visit			
No revisit	298 (71.9)	116 (28.1)	0.035*
1–2	92 (83.6)	18 (16.4)	
≤ 3	88 (70.9)	26 (29.1)	

* P-value less than 0.05 was determined significant, N, number

The statistical analysis indicated that, except for educational level, the other demographic variables including gender, age, marital status and occupation did not show a significant association with the number of revisits (Table 4). Among the patients with a bachelor's degree or above, the proportion of patients with no revisits (after the first visit) was significantly lower (52.5%), while the ratio of treatment dropout was higher for patients with lower education levels (64.7% of illiterates, 66% of patients with elementary and secondary education, and 70.6% of patients with diploma or above) (P = 0.014).

**Table 4:** Factors associated with the number of revisits

Variable	No revisit (%)	1–2 (%)	≤ 3 (%)	P-value*
Gender				
Male	125 (65.1)	32 (16.6)	35 (18.3)	0.975
Female	306 (65.1)	81 (17.2)	83 (17.7)	
Age				
0–17	122 (65.2)	33 (17.6)	32 (17.2)	0.819
18–39	193 (67.2)	47 (16.4)	47 (16.4)	
40–59	96 (61.5)	30 (19.2)	30 (19.2)	
60 ≤	20 (52.6)	9 (23.7)	9 (23.7)	
Marital status				
Married	242 (65.9)	58 (15.8)	67 (18.3)	0.366
Single	32 (64)	8 (16)	10 (20)	
Widowed	18 (75)	4 (16.6)	2 (8.4)	
Divorced	17 (50)	10 (29.4)	7 (20.1)	
Education				
Illiterate	22 (64.7)	2 (5.8)	10 (29.5)	0.014*
Elementary and secondary	111 (66)	25 (14.9)	32 (19.1)	
Diploma or above diploma	108 (70.6)	29 (18.9)	16 (10.5)	
Bachelor or above	32 (52.5)	13 (21.3)	16 (26.2)	
Occupation				
Housewife and unemployed	178 (62)	52 (18.1)	57 (19.9)	0.417
Student	9 (81.8)	2 (18.2)	0	
Self-employment	33 (71.7)	8 (17.4)	5 (10.9)	
Worker	30 (68.2)	4 (9.1)	10 (22.7)	
Government employee	18 (56.3)	6 (18.7)	8 (25)	

* P-value less than 0.05 was determined significant, N, number

## Discussion

The high cost of treating patients with mental health conditions and the chronic nature of many illnesses increase the cost of health services [19]. Previous research has shown that outpatient treatment can reduce the cost of mental health care and minimize recurring hospitalizations when compared to hospitalization [19, 20]. For example In Iran, 18% of the health organization's budget is spent on mental health problems in mental hospitals, while patients with a history of admission in psychiatric hospitals are the minority of people with mental health condition [21]. CMHC as a specialized outpatient providing mental health care was found to have positive impacts on the cost effectiveness and efficacy of in Iran and other countries [15, 17, 22, 23]. Five years after the establishment of CMHC in Iran, Sharifi et al. suggested that skilled staff, a better source of funding, and more integration into the healthcare system can enhance the performance and efficacy of CMHC [17].

Investigating the epidemiological factors in patients with mental health conditions at the CMHC can indeed provide valuable insights for health policymakers in Iran. By understanding the patterns and characteristics of these patients, policymakers can make more informed decisions regarding the allocation of resources, development of targeted interventions, and improvement of mental health services in CMHCs [23]. Majority of patients entered in this study were female (71% vs 29%), This finding is consistent with previous studies in Iran and other countries, which have shown that mental health conditions are more prevalent in women than in men[2, 12, 19, 24, 25]. It seems possible that women are more susceptible to mental illnesses due to a lack of social support or biological factors. [4, 26]. Another potential explanation for this gender disparity is that pregnant patients mainly seek care at PHC and referral system in Iran [27], although this study did not specifically examine the rate of pregnant female patients. Majority of patients in this study (Table 1) were housewives (56.4%) and unemployed (11.9%). A higher unemployment rate is expected for patients with mental illness than those without mental illness [28]. In this study, women who have no work outside the home (unemployed) and instead do housework were considered housewives. Mental illnesses may be more common among housewives [28]. The CMHC in Mashhad provides mental health services from 8 am to 2 pm. However, many employed individuals are unable to attend during these hours due to work commitments. These may explain why a majority of our patients are those who have greater availability in the morning, such as housewives and the unemployed. Also, specialized mental health services at the CMHC are more affordable compared private inpatient mental health clinics especially for unemployed individuals [15, 17]. In current study, most patients were between 18 and 39 years old (44.8%) (Table 1) this finding was similar to another study in Yazd's CMHC, a city in central part of Iran [16]. However, a nationwide evaluation demonstrated that mental illness occurs more frequently in patients between 40 and 55 in Iran [29].

MDD was identified as the most prevalent disorder among all patients, followed by GAD Similarly, a national survey conducted in Iran found that most patients with a history of mental illnesses had symptoms related to depression and anxiety [30]. Similarly, depression and anxiety are the most common mental illnesses in Qatar, Germany and southern Asian countries [24, 31, 32]. In children, ADHD, GAD, and ODD were more prevalent. Similarly, A study assessing the prevalence of mental illness in high-income countries found that anxiety disorders, ADHD, and ODD were more common among children [33], in another systematic review evaluating the mental disorders in children between 1 and 7 years revealed that ODD and ADHD were the most common mental illness [34]. In other studies conducted in Iran, somatization disorder was found to be one of the most common types of mental illnesses [2, 4]. However, in our study, somatization disorders were less prevalent compared to these previous findings (Table 2). One possible explanation for this difference could be related to the data collection method, as some patients had multiple mental illnesses but only the primary diagnosis was recorded in our study. Additionally, somatization disorders might be underdiagnosed in outpatient health services in Iran, as we found that among middle-aged patients at PHCs in Khorasan Razavi province found a lower prevalence of somatoform disorder than expected [35].

The majority of patients (74.9%) visited the psychiatrist through the referral system. Female gender, patients with lower education level, unemployed and housewives were associated with more referral health system (PHCs or Psychiatric hospitals) (Table 3). This suggests that the patients with lower socioeconomic conditions were referred more through health system to Mashhad ‘s CMHC. Studies investigated that PHCs can assist in the screening, treatment, and referral of common mental disorders (such as depression) and even some less common but more serious mental disorders (such as psychosis), especially for groups of people that do not have access to specialized mental health services [35–37]. Patients' lack of awareness about healthcare services may lead them to bypass the referral system, causing delays in treatment and lower-quality healthcare facilities [38, 39]. Therefore, a good referral system can facilitate the achievement of CMHC's goals and new strategies are required to ensure equitable access to mental health services for all individuals is vital.

Fernández D et al. revealed that treatment dropout occurs in the primary sessions is mainly expected; in our study, 65% of patients had only one appointment with a psychiatrist [12]. A similar study conducted among Tehran's CMHC patients revealed that only 41% of the subjects demonstrated compliance with prescribed follow-up protocols [40]. Some studies have shown that patients with higher education levels are less likely to experience treatment dropout just like our study (Table 4) [12, 41]. However, there was no association between patient referring to Tehran’s CMHC demographics and treatment dropout [42]. Our study was limited to examining factors influencing treatment discontinuation, except patient-related factors such as severity of mental illness and physicians’ and health care providers’ characteristics (e.g., empathy) [43]. The probable reason behind it is that educated participants are more informed about mental health treatment and can be associated with higher mental health literacy. Understanding the treatment dropout rates among patients with mental health conditions in the CMHC can guide the development of strategies to enhance treatment engagement and retention, particularly in patients with lower education level.

### Limitations

This study suffers from the limitation that most medical records were excluded from this investigation. It should be noted that some patients were referred back to PHCs or psychiatric hospitals after starting treatment, but this information was not documented in the center's medical records. Additionally, our research focused on patient revisits, regardless of treatment completion, which differs from treatment dropout and should be interpreted with caution.

## Conclusions

It has been about two decades since the first CMHC went into operation. These centers play an essential role in diagnosing and treating non-hospitalized patients with mental health condition in Iran with lower prices for the health care system. We presented epidemiological aspects of patients with mental illnesses at CMCH in Mashhad and the capacity of this center. The study found that other parts of the health care system, especially the PHCs, should be more diligent in screening and referring patients with mental health condition, especially about women with lower socioeconomic levels. This study suggests that to achieve higher levels of mental health in society, CMHC should prioritize screening for common psychiatric disorders such as MDD and GAD. Furthermore, enhancing healthcare services in CMHCs can promote continued treatment adherence. Overall, further research on these topics can help address gaps and challenges in mental health services, guiding evidence-based policies and interventions in Iran.

## Data Availability

The data supporting this study's findings are available from the corresponding author upon request. However, the permission of The Ethical Committee of Mashhad University of Medical Sciences is necessary.

## Declarations

All methods were carried out by relevant guidelines and regulations.
